# Absence of Vitamin K-Dependent γ-Carboxylation in Human Periostin Extracted from Fibrotic Lung or Secreted from a Cell Line Engineered to Optimize γ-Carboxylation

**DOI:** 10.1371/journal.pone.0135374

**Published:** 2015-08-14

**Authors:** Douglas S. Annis, Hanqing Ma, Danika M. Balas, Kraig T. Kumfer, Nathan Sandbo, Gregory K. Potts, Joshua J. Coon, Deane F. Mosher

**Affiliations:** 1 Department of Biomolecular Chemistry, University of Wisconsin-Madison, Madison, Wisconsin, United States of America; 2 Morgridge Institute for Research, University of Wisconsin-Madison, Madison, Wisconsin, United States of America; 3 Department of Medicine, University of Wisconsin-Madison, Madison, Wisconsin, United States of America; 4 Department of Chemistry, University of Wisconsin-Madison, Madison, Wisconsin, United States of America; IIBB-CSIC-IDIBAPS, SPAIN

## Abstract

Periostin (PN, gene name POSTN) is an extracellular matrix protein that is up-regulated in bronchial epithelial cells and lung fibroblasts by TH-2 cytokines. Its paralog, TGF-β-induced protein (βig-h3, gene name TGFBI), is also expressed in the lung and up-regulated in bronchial myofibroblasts by TGF-β. PN and βig-h3 contain fasciclin 1 modules that harbor putative recognition sequences for γ-glutamyl carboxylase and are annotated in UniProt as undergoing vitamin K-dependent γ-carboxylation of multiple glutamic acid residues. γ-carboxylation profoundly alters activities of other proteins subject to the modification, e.g., blood coagulation factors, and would be expected to alter the structure and function of PN and βig-h3. To analyze for the presence of γ-carboxylation, proteins extracted from fibrotic lung were reacted with monoclonal antibodies specific for PN, βig-h3, or modification with γ-carboxyglutamic acid (Gla). In Western blots of 1-dimensional gels, bands stained with anti-PN or -βig-h3 did not match those stained with anti-Gla. In 2-dimensional gels, anti-PN-positive spots had pIs of 7.0 to >8, as expected for the unmodified protein, and there was no overlap between anti-PN-positive and anti-Gla-positive spots. Recombinant PN and blood coagulation factor VII were produced in HEK293 cells that had been transfected with vitamin K 2, 3-epoxide reductase C1 to optimize γ-carboxylation. Recombinant PN secreted from these cells did not react with anti-Gla antibody and had pIs similar to that found in extracts of fibrotic lung whereas secreted factor VII reacted strongly with anti-Gla antibody. Over 67% coverage of recombinant PN was achieved by mass spectrometry, including peptides with 19 of the 24 glutamates considered targets of γ-carboxylation, but analysis revealed no modification. Over 86% sequence coverage and three modified glutamic acid residues were identified in recombinant fVII. These data indicate that PN and βig-h3 are not subject to vitamin K-dependent γ-carboxylation.

## Introduction

The extracellular matrix (ECM) proteins periostin (PN, gene name POSTN) and TGF-β-induced protein (βig-h3, gene name TGFBI) were discovered in the early 1990s [1, 2]. Nearly two decades later, Coutu et al. described evidence that these proteins are modified by γ-carboxylation [3]. γ-carboxylation is a vitamin K-dependent post-translational modification that has profound effects on protein structure-function, e.g., vitamin K-dependent blood coagulation factors or the ECM proteins matrix-Gla protein and osteocalcin [4, 5]. γ-carboxylation should have similarly important effects on the structure and function of PN and βig-h3. The original goal of the present study was to describe these effects; however, the study evolved into a re-analysis of whether the proteins are indeed γ-carboxylated.

PN and βig-h3 vary most strikingly in their C-terminal tails [6]. These proteins have a similar domain structure for the remainder of the molecule, with each containing a single N-terminal cysteine-rich EMI domain [7, 8], followed by 4 tandem fasciclin 1 (FAS1) modules [2, 9]. The FAS1 modules of PN and βig-h3 harbor putative recognition sequences for γ-glutamyl carboxylase. This feature of the modules was recognized when it was found that 2-dimensional isoelectric focusing/electrophoresis in sodium dodecyl sulfate (SDS) of proteins secreted by cultured mouse mesenchymal stromal cells resolved acidic proteins of the size of PN and βig-h3 that reacted with monoclonal antibody (mAb) specific for the γ-carboxyl-glutamic acid (Gla) modification and yielded peptides from PN and βig-h3 when excised, trypsinized, and analyzed by mass spectrometry [3]. Based on these observations and putative carboxylase recognition sequences in 3 of the 4 FAS1 modules of both proteins, UniProt as queried on May 5, 2015, annotates PN ([Q15063-POSTN_HUMAN]) as containing up to 24 Gla residues, and βig-h3 ([Q15582-BGH3_HUMAN]) up to 29 Gla residues. To our knowledge, no further investigations of γ-carboxylation of PN or Big-h3, *e*.*g*., the actual numbers of Gla residues in these proteins, have been attempted or published.

We took a 2-pronged approach to the study of γ-carboxylation of human PN and βig-h3. Periostin is up-regulated in bronchial epithelial cells and lung fibroblasts by TH-2 cytokines IL-4 and IL-13 and deposited in airway of asthmatic subjects, of rodents provoked to model the inflammation of asthma [10–14], and of patients with idiopathic lung fibrosis [15]. βig-h3 is expressed in the lung and up-regulated in bronchial myofibroblasts by TGF-β [16]. To determine if the proteins deposited in ECM are γ-carboxylated, we extracted proteins from normal or fibrotic lung and analyzed for reaction with Gla-specific antibody and acidic shift in isoelectric point due to introduction of extra carboxyl groups. We also attempted to produce recombinant γ-carboxylated PN in HEK293 cells that had been transfected with VKOR (vitamin K 2, 3-epoxide reductase C1) for the purpose of increasing the yield of γ-carboxylated vitamin K-dependent proteins. We now report that, despite using reagents and methods similar to those that led Coutu et al. [3] to conclude that PN and βig-h3 are γ-carboxylated, we find no evidence for the modification.

## Materials and Methods

### Cell lines

The HEK293VKOR cell line is HEK293 (ATCC) that had been transfected with the plasmid pIRESpuro3-VKOR encoding vitamin K 2, 3-epoxide reductase C1 (VKOR) by Dr. Darrel Stafford, University of North Carolina [17] and was a gift from Dr. James Morrissey, University of Illinois.

### Proteins

Factor II (fII) and factor IX (fIX) were from Haematologic Technologies, Inc, (Essex Junction, VT). Protein S was a gift from Drs. Jose Fernandez and John Griffin, The Scripps Research Institute, La Jolla, CA.

### Antibodies

The antibodies used are as follows: PN, murine monoclonal (mAb) Stiny-1 (Adipogen, San Diego, CA) and rabbit polyclonal anti-PN ab14041 (Abcam, Cambridge, MA); βig-h3, murine mAb βig-h3 (Proteintech, Chicago, IL); γ-carboxyglutamyl (Gla) residues independent of protein context, murine mAb 3570 [18](Sekisui Diagnostics, Lexington, MA); fII, sheep polyclonal antibodies (Haematologic Technologies, Inc, Essex Junction, VT); and peroxidase conjugated anti-mouse, anti-Sheep, and anti-rabbit IgG (Jackson ImmunoResearch, West Grove, PA).

### Ethics Statement

De-identified tissue samples were obtained from lung transplants or thoracic surgical resection specimens obtained by the Carbone Cancer Center Translational Science BioCore at the University of Wisconsin, Madison, under IRB approval #2011–0840.

### Preparation of lung extracts

Lung samples were kept frozen with liquid nitrogen while grinding to a fine powder with a mortar and pestle. Proteins were extracted from this powder using urea/EDTA (8M urea, 10mM Tris, 150mM NaCl, 2mM EDTA, pH7.4) or SDS (5% SDS, 10% glycerol, 60mM Tris, pH6.8) at a ratio of 100 μl extraction buffer per 10mg tissue. Powdered tissue was mixed with extraction buffer, incubated for 30 or 60 min at ambient temperature followed by centrifugation at 16000g for 15 min to remove insoluble material.

### Two-Dimensional Electrophoresis

Two-dimensional electrophoresis was performed according to the carrier ampholine method of isoelectric focusing [19, 20] by Kendrick Labs, Inc. (Madison, WI). Briefly, isoelectric focusing was carried out using 2% pH 3–10 Isodalt Servalytes (Serva, Heidelberg, Germany) for 9600 volt-hrs. One μg of tropomyosin (pI 5.2) was used as an internal standard. The focused proteins were then reduced, and the tube gel overlaid onto an 8 or 10% SDS PAGE gel. After electrophoresis the gel was transferred to PVDF membrane, stained with Coomassie Brilliant Blue R-250, documented, and sent to our lab where it was probed in Western blot.

### Western blot

Lung extracts, conditioned cell media or purified proteins were resolved on SDS-PAGE gels in reducing conditions then transferred to PVDF membranes. Primary antibodies were diluted as follows; mouse mAb anti-PN, mouse mAb anti-Gla, and rabbit anti-PN were used at 0.5 μg/mL. Anti-βig-h3 mAb was diluted to 2.0 μg/mL, and sheep anti-fII was diluted 1/10,000. Positive bands were detected by species- and isotype-specific peroxidase-conjugated antibodies (1/20,000), and enhanced chemiluminescence technology (Perkin Elmer, Waltham, MA). Specificity of the secondary antibodies was assessed by omitting the primary antibodies.

### Protein Expression

#### Cloning of PN

PN cDNA was obtained by reverse transcription of RNA isolated from MG-63 cells using M-MLV reverse transcriptase (Promega, Madison, WI) and a PN-specific oligonucleotide (5’ TTT GGA TTT TCA CTG AGA AC 3’) coding for a region at the end of the mature protein. The cDNA was used as template for polymerase chain reaction (PCR) and PN insert DNA was cloned into either pcDNA3.1+ (Invitrogen, Carlsbad, CA) or pAcGP67.coco [21] using standard molecular biology techniques. The complete insert sequence was verified before expression.

#### Expression in mammalian cells

Human PN containing EMI and the four FAS1 but lacking variably spliced C-terminal sequences encoded by exons 17, 18, 19, and 21 ([POSTN_HUMAN isoform 7; UniProt: Q15063-7]) was produced as a secreted protein in HEK293VKOR cells. Briefly, human PN isoform 7 (PN0) in pcDNA3.1+ (Invitrogen, Carlsbad, CA), was transfected into HEK293VKOR using FuGENE6 transfection reagent (Promega, Madison, WI). Stable transfectants were grown in DMEM supplemented with 10% fetal bovine serum, and selection antibiotics puromycin (1.75 μg/mL) (EMD Millipore, Billerica, MA) and G418 (450 μg/mL)(Hyclone Labs, Logan, UT). For serum free expression, cells were plated in serum containing media plus 10 μg/mL vitamin K_1_ (Sigma Aldrich, St Louis, MO) and grown to 80% confluence, after which the cells were gently washed twice with PBS and serum free DMEM/F-12, 50/50 with L-glutamine (Mediatech, Manassas, VA) media supplemented with selection antibiotics, 1x ITS (Mediatech, Manassas, VA) and 10 μg/mL vitamin K_1_ was added to the plates. Conditioned media was collected and replaced with fresh on day 3 and collected again on day 7. Secreted PN0, which has a C-terminal thrombin-cleavage site followed by hexa-histidine tag, was purified by Ni-chelate chromatography in the presence of 6M urea. PN0 has a calculated molecular weight of 79.9 kDa. In SDS-PAGE, PN0 separated into a monomer of ~76 kDa and lesser amounts of an apparent dimer of ~162 kDa without reduction and a monomer of ~78 kDa with reduction (S1 Fig).

Factor VII harboring the R152A (R15A by chymotrypsin numbering) mutation was produced as a secreted protein in HEK293VKOR cells and is referred to hereafter merely as factor VII or fVII. The cDNA was cloned into pcDNA3.1(+)(Invitrogen, Carlsbad, CA) and transfected into HEK293VKOR cells using FuGENE6 (Promega, Madison, WI). Stable transfectants were grown in DMEM/F12, 50/50 (Mediatech, Manassas, VA) supplemented with 10% FBS, 5ug/mL vitamin K_1_ (Sigma Aldrich, St. Louis, MO), 1.75ug/mL puromycin (EMD Millipore, Billerica, MA), and 400 μg/mL G418 (RPI, Mount Prospect, IL). After growth to 80% confluence, cells were washed with HBSS (Mediatech, Manassas, VA) and media replaced with DMEM/F12, 50/50 with L-glutamine (Mediatech, Manassas, VA) supplemented with 1x ITS (Mediatech, Manassas, VA), 5 μg/mL vitamin K_1_, 200 μg/mL G418 and 1.75 μg/mL puromycin every 2–3 days until cell confluence was <10%. This serum-free media was concentrated ~10-fold with a 30kDa MWCO filter (EMD Millipore, Billerica, MA) in a stirred cell concentrator and purified by serial column chromatography with the calcium-dependent anti-fVII antibody 1150 [22], then with the anion exchange resin monoQ (GE Healthcare Life Sciences, Pittsburgh, PA). The calcium-dependent antibody recognizes fVII regardless of γ-carboxylation status (unpublished observation). Homogeneity was confirmed by single bands on silver-staining of SDS-PAGE (S2 Fig). Polyethelene glycol (PEG) 8000, 0.1%, was added to fVII for stabilization.

#### Expression with Baculovirus

The N-terminal region of fibronectin, FN70[23], and PN in pAcGP67.coco were expressed and purified as previously described [21, 24]. The medium used for expression did not contain vitamin K.

### Mass Spectrometry

#### Searching Methods for Deposited Data

Data from a published mass spectrometric analysis of SDS-insoluble fractions of metastatic tumor samples, deposited by Naba et al. [25] in the public proteomics repository MASSive (http://massive.ucsd.edu) under the identifier MSV000078535, were downloaded to search for potential carboxylation modifications using OMSSA within the COMPASS software suite [26]. Mass spectra were searched with a 125 ppm precursor mass tolerance and 0.35 Da fragment tolerances for b and y ions produced CAD MS/MS fragmentation. Additionally, these peptide spectra were searched with static carbamidomethylation of cysteine residues and dynamic oxidation of methionine residues and carboxylation (+44 Da) of glutamic acid or aspartic acid residues.

#### ETD and AI-ETD for γ-carboxylation localization with PN and fVII

Purified protein samples of PN0 (12.9 μg) and fVII (56 μg) were precipitated in a solution of 90% MeOH. FVII samples were washed 7 times with 100% methanol to remove PEG 8000. Samples were suspended in 8 M urea, 40 mM chloroacetamide, and 10 mM TCEP to denature, reduce, and alkylate proteins. The single PN0 and fVII samples were each split in half, and each was diluted to 1.5 M urea using 50 mM Tris. Each of the two resulting PN0 and fVII samples were digested with trypsin or chymotrypsin overnight at room temperature. The tryptic and chymotryptic PN0 and fVII peptide samples were each desalted using Strata-X columns, dried down, and resuspended in 0.2% formic acid. Each of the four samples was separated using a 90 minute nano-liquid chromatography gradient with in-stream analysis by an Oribtrap Elite mass spectrometer (Thermo Scientific, Waltham, MA) equipped with a multi-dissociation cell (MDC) [27]. A data dependent top 10 mass spectrometry method was designed to collect a 60,000 resolving power MS^1^ scan followed by 10 subsequent 15,000 resolving power MS/MS scans using either Electron Transfer Dissociation (ETD) or Activated Ion ETD (AI-ETD) for peptide fragmentation [27, 28]. ETD or AI-ETD fragmentation utilized an Automatic Gain Control (AGC) target value of 4.0 x 10^5^ and 200 msec maximum injection times for MS/MS scans. Fluoranthene reagent anions were accumulated for 15 msec in the instrument’s MDC.

PN0 and fVII data were searched using [26] Proteome Discoverer 1.4.0.228 with the Sequest search algorithm. Thermo RAW files were searched against a Homo sapiens target-decoy database (Uniprot, downloaded 11/06/2014) which included PN0 and coagulation factor VII (R152A mutant). Both PN0 and fVII datasets were searched using a 50 ppm precursor mass tolerance and 0.02 Da fragment tolerance for c and z ions produced by ETD and AI-ETD fragmentation. The chymotryptic and tryptic samples were searched with their appropriate enzymes, along with static carbadimomethyl cysteine and dynamic methionine oxidation and γ-carboxylation modifications. Resulting peptide identifications were filtered to 1% false discovery rate (FDR). Raw data are available at FigShare (http://figshare.com/) using the following link: http://dx.doi.org/10.6084/m9.figshare.1497978.

## Results

### Detection of PN and βig-h3 extracted from lung

Increased amounts of PN are deposited in lung tissues of patients with idiopathic pulmonary fibrosis as assessed by immunocytochemistry [15]. It is not known if the deposited protein is modified by vitamin K-dependent γ-carboxylation. To determine this, tissue samples from fibrotic and normal lung were pulverized, and proteins extracted with SDS or urea/EDTA, and analyzed by Western blotting looking for congruent bands that react with anti-PN mAb and the same anti-Gla mAb used to demonstrate γ-carboxylation by Coutu et al. [3].

**Fig 1:**
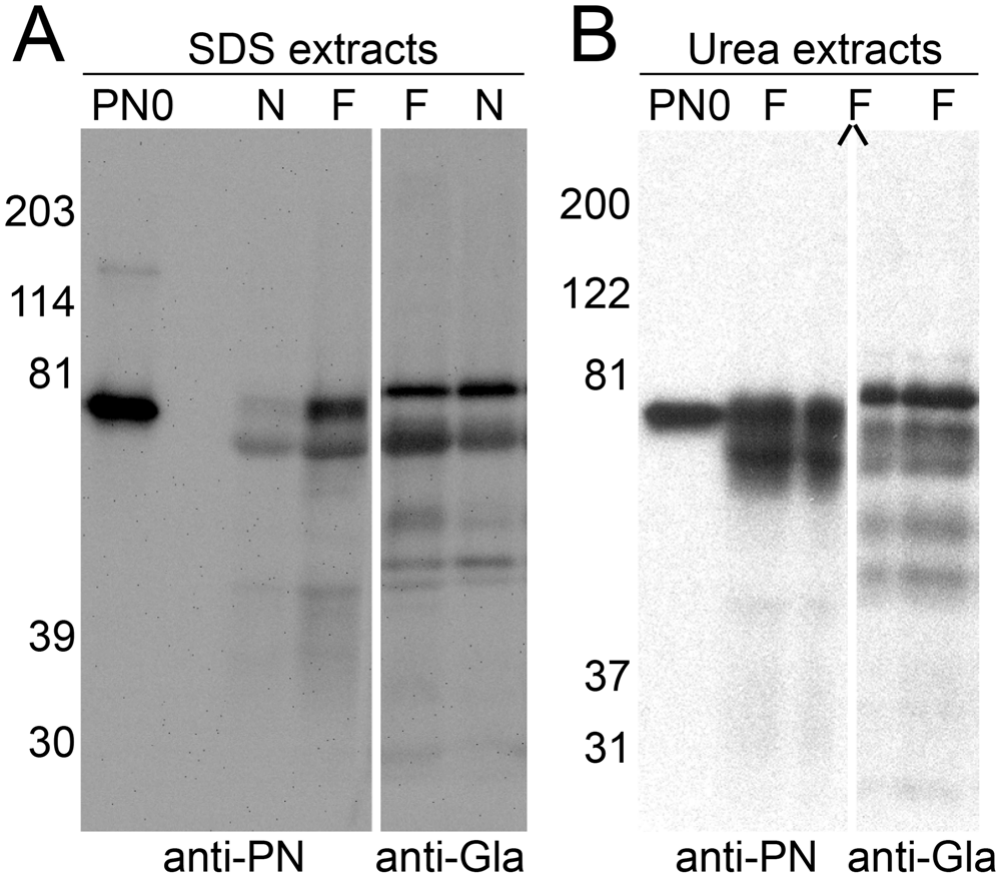
Fibrotic lung extracts have increased amounts of PN, and alignment of PN-positive and Gla-positive bands is inconclusive. (A) A representative Western blot of proteins extracted with SDS from pulverized lung tissue (10mg tissue per 0.1 mL extraction buffer). SDS extracts (2.5 μl/lane) of fibrotic (F) and normal (N) lung sample, were blotted with mouse anti-PN (left) and anti-Gla (right). PN-positive bands in lung extracts migrate at approximately 79 kDa, similar to the migration of PN0 expressed in HEK293VKOR cells (PN0). (B) Urea/EDTA extracts (10.0 μl/lane) of fibrotic lung (F) have protein patterns similar to those seen with the SDS extracts. A lane has been split (side slashes) and probed with both anti-PN (left) and anti-Gla (right) antibodies. The anti-PN- and anti-Gla-positive bands do not align exactly but bands do overlap. PN0 was expressed in insect cells with baculovirus.

Western blot analysis using anti-PN mAb demonstrated increased density in extracts of fibrotic versus normal lung of a band that migrated similarly to recombinant PN0 (Fig 1A). PN0 is the smallest of the PN splice variants, migrates in SDS-PAGE at ~78kDa, and has a calculated mass of 79.9kDa. A second band slightly below the PN band was variably found in control blots in which the primary antibody was omitted and likely represents cross-reactivity of the secondary goat anti-mouse IgG conjugate with albumin in the extract. Anti-Gla recognized 4–5 bands specifically, including 2 close in migration to the band that stained specifically with anti-PN. These bands did not align exactly, however, and when a single lane was split in half, each half was blotted separately with anti-PN or anti-Gla, and the two halves were matched prior to imaging, the band that stained specifically with anti-PN was slightly below the most slowly migrating band that stained specifically with anti-Gla (Fig 1B).

**Fig 2:**
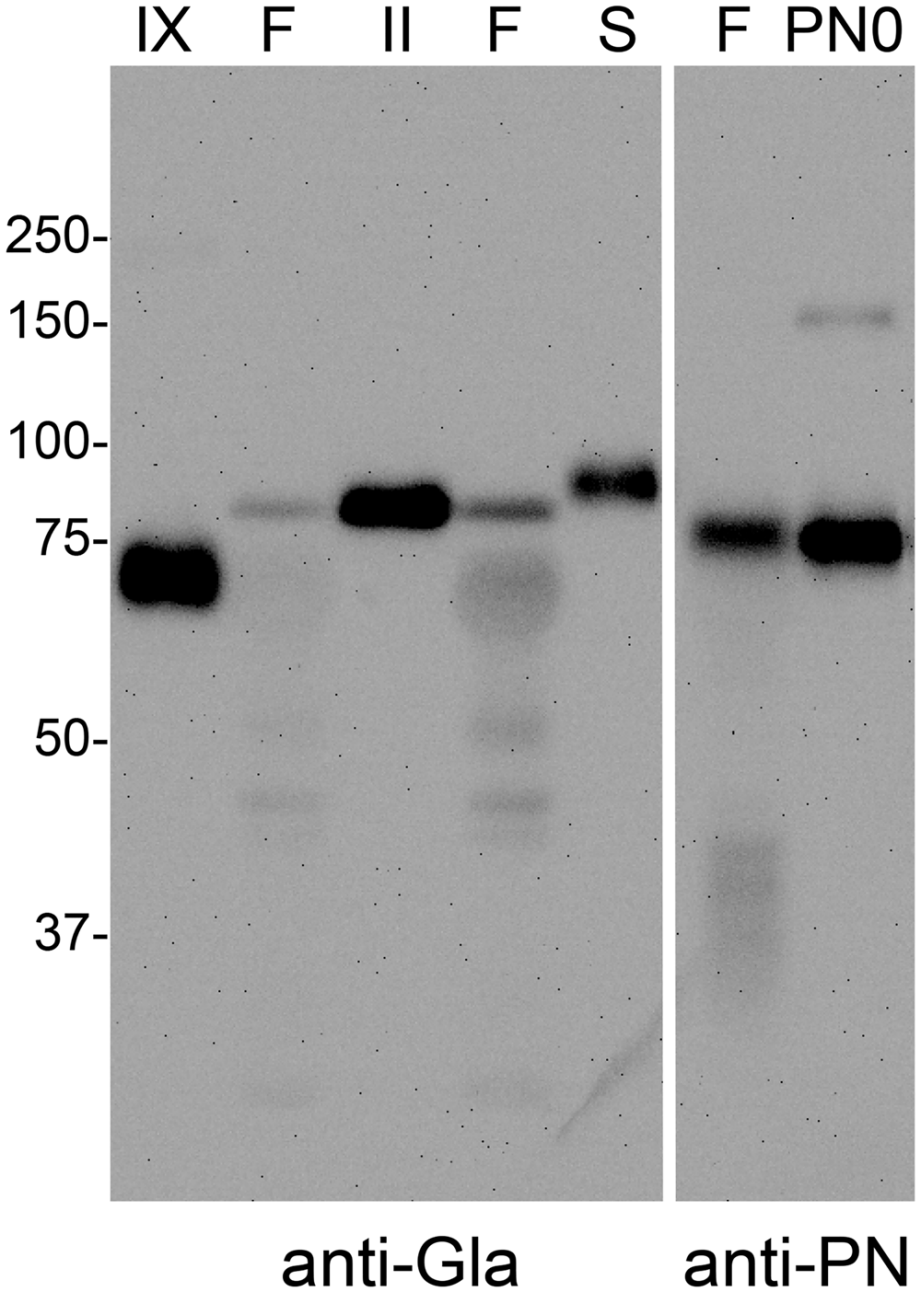
Gla positive bands in fibrotic lung extracts co-migrate with fII and fIX. SDS-extracts of fibrotic lung (F) and purified fIX (IX), fII (II), and protein S (S) were immunoblotted with anti-Gla (left). Gla positive bands in fibrotic lung extracts have similar migration and band shape as fII and fIX. Protein S migrated slightly slower than fII. Periostin from fibrotic lung extracts is detected in a 79 kDa band between fIX and fII, and has a similar migration to recombinant PN0 produced in HEK293VKOR cells (PN0) when stained with anti-PN (right).

We reasoned that the anti-Gla-staining bands may represent, in part, staining of vitamin-K dependent plasma proteins present in lung vasculature or interstitial fluid. We therefore ran standards of plasma-derived human fII, protein S, and fIX alongside the fibrotic lung extract and blotted with anti-Gla. All 3 proteins reacted strongly with anti-Gla. Bands in the fII and fIX samples co-aligned, respectively, with Gla-positive bands of 84 kDa and a diffuse band of 67 to 77kDa in the lung extract (Fig 2). Protein S had a slightly slower migration than fII and did not line up with an anti-Gla staining band in the extract. A parallel blot with anti-PN revealed a band that co-migrated with PN0 and slightly faster than the fII standard or the 84 kDa band in the extract that reacted with anti-Gla (Fig 2). Western blots with fII specific antibodies detected a band of 84kDa in fibrotic lung extracts (S3 Fig). Based on these results, we conclude that the Gla-positive 84kDa band in lung extracts represents fII.

**Fig 3:**
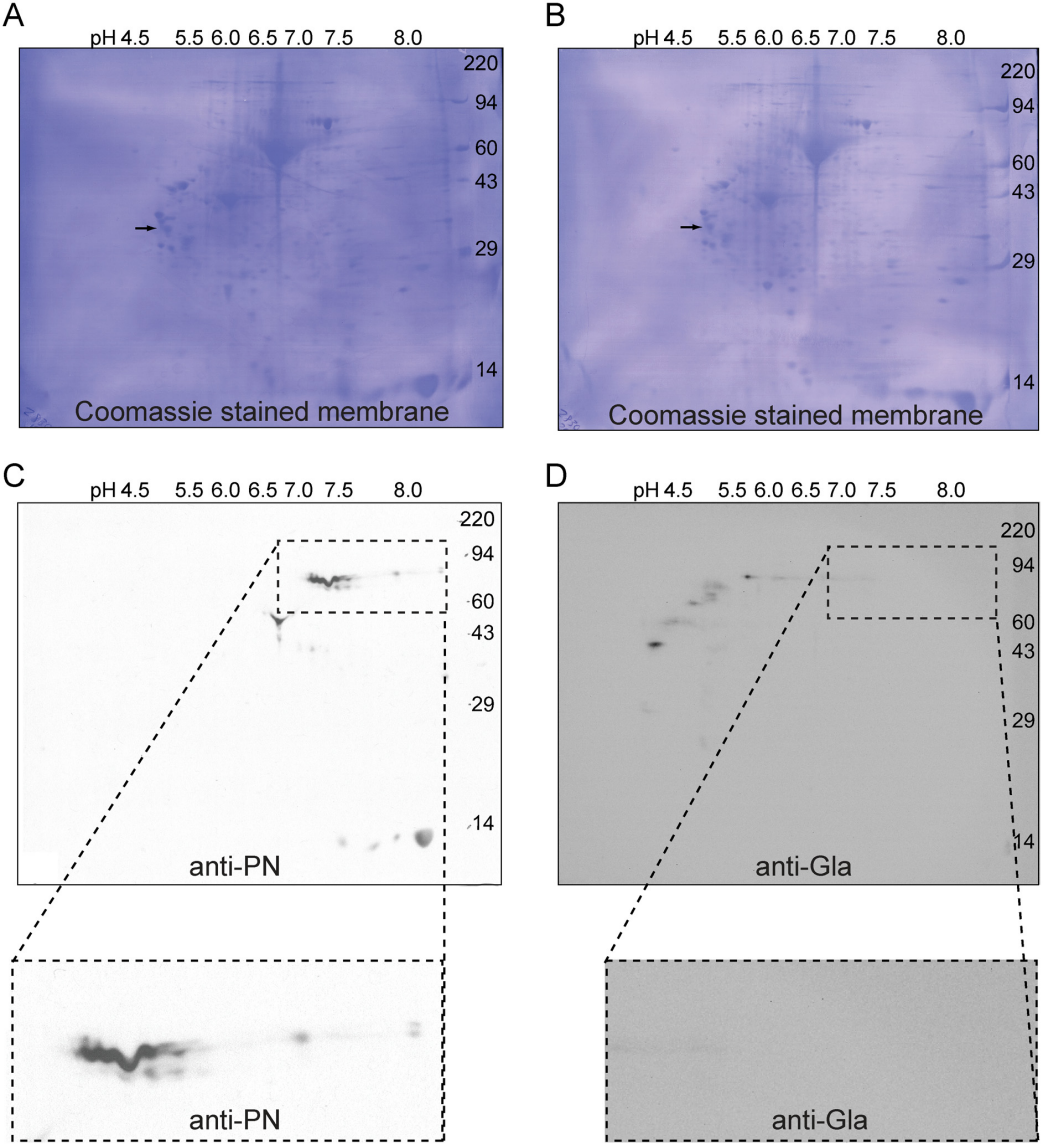
PN extracted from fibrotic human lung has the pI expected of un-modified protein and does not react with anti-Gla. Approximately 200 μg of protein extracted with SDS from fibrotic lung samples were subjected in parallel to 2-dimensional isoelectric focusing/electrophoresis, transferred to PVDF membranes, and Western blotted. The membranes were stained with Coomassie blue (A and B) and probed with (C) mouse anti-PN or (D) mouse anti-Gla mAbs. Enlarged views of the PN-positive region are shown in the insets for both blots. Isoelectric points of PN-positive spots ranged between pH 7.0 and > pH 8.0. Gla-positive spots were more acidic and did not overlap with PN positive spots. An internal isoelectric focusing standard, tropomyosin, migrated as a doublet with the lower polypeptide spot (arrows) of MW 33 kDa and pI 5.2 on the parallel 10% gels.

Were PN modified by γ-carboxylation, each Gla modification would be expected to lower its isoelectric point (pI) due to addition of a carboxyl group. In UniProt, PN is annotated to have 24 potential Gla modifications. Isoelectric point calculations of the un-carboxylated splice variants of PN by the Expasy PI/Mw Tool (http://web.expasy.org/compute_pi/) ranged between a variant that includes three of the alternatively spliced exons ([POSTN_HUMAN isoform 5; UniProt: Q15063-5]) with pI of 7.08 and a variant which includes one of the exons ([POSTN_HUMAN isoform 4; Uniprot: Q15063-4]) with pI 8.40. Addition of 24 Gla modifications was calculated to change the pIs to 4.5 to 5.5. To look for this acidic shift in pI and allow for a more critical comparison of PN- and Gla-positive bands, proteins extracted with SDS from fibrotic lung samples were subjected to 2-dimensional isoelectric focusing/electrophoresis and blotted in parallel with monoclonal anti-PN and anti-Gla mAbs (Fig 3). Coomassie blue staining of the membranes prior to blotting demonstrated similar protein patterns (Fig 3A and 3B). PN-positive spots were detected between 73 and 79 kDa (Fig 3C), consistent with migration observed with recombinant PN in reducing conditions and the predicted range of molecular weights of the PN splice variants. The isoelectric points of PN-positive spots ranged between pH 7.0 and > pH 8.0, which are the calculated pIs of the various splice variants of un-γ-carboxylated PN. Anti-Gla identified an array of spots with sizes ranging from approximately 87 to 47 kDa and pIs ranging from 4.0 to 6.0; these spots were far away from the PN-positive spots (Fig 3D). To address the possibility that the epitope of the anti-PN monoclonal is lost upon γ-carboxylation and to explore the nature of the unexpected”Y” shaped spot (~50 kDa with pI between pH 6.5 and 7.0) that stained with anti-PN mAb (Fig 3C), immunoblotting was also done with polyclonal antibodies to PN. The pattern was similar to what was found with the mAb to PN except for the appearance of several low mw spots in the basic range, one low mw spot in the acidic range, and that the”Y” shaped spot was not seen (S4 Fig). All PN-positive spots were negative with the anti-Gla antibody (Fig 3). FII and fIX, which have pIs of 4.7–4.9, and 4.0–4.5 [29], respectively, presumably account for acidic Gla-positive spots with apparent sizes similar to PN. We have not done further analysis to identify these and the other Gla-staining spots definitively.

**Fig 4:**
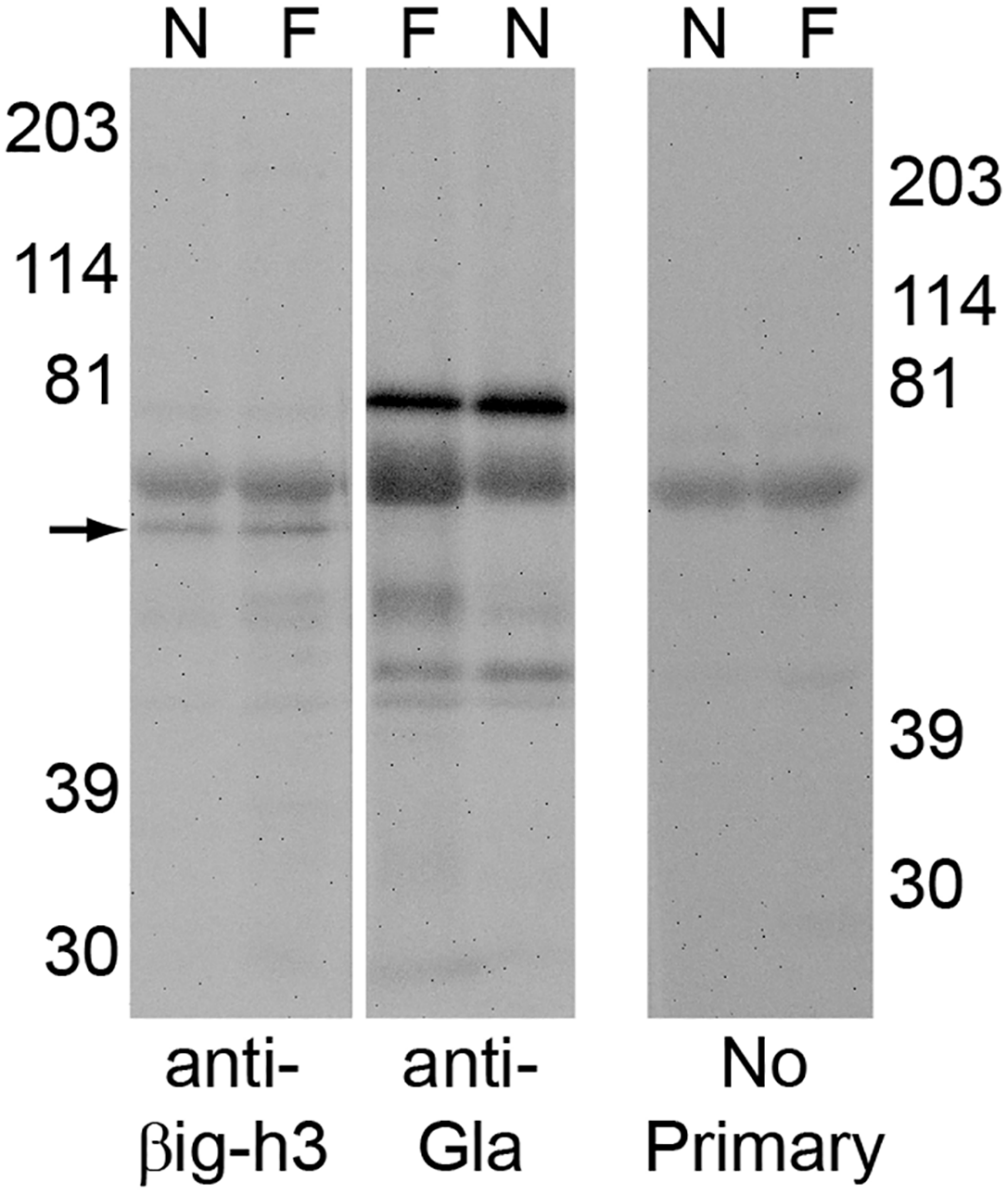
βig-h3 extracted from lung does not react with anti-Gla. Pulverized fibrotic (F) and normal (N) lung samples were extracted with SDS and probed with anti-βig-h3 (left) and anti-Gla (middle). βig-h3 positive bands (arrow) were observed in both normal and fibrotic lung extractions. The βig-h3 band was not detected with the anti-Gla antibody. A duplicate blot, from a different gel, had primary antibody omitted (right), and shows a band attributed to the non-specific binding of the secondary antibody to the lung extracts. This band is seen in both the anti-βig-h3 (left), and the anti-Gla (middle) blots.

Like PN, βig-h3 is an ECM protein with FAS1 modules that harbor putative recognition sequences for γ-glutamyl carboxylase [3]. The pulverized and SDS-extracted normal and fibrotic lung samples were immunoblotted for the presence of βig-h3 and Gla residues. Somewhat surprisingly, given published evidence that βig-h3 is up-regulated in the cultured bronchial myofibroblasts by TGFβ, a pro-fibrotic cytokine [16], anti-βig-h3-positive bands of similar intensity were detected by Western blotting in both samples. No anti-Gla-positive band was present at this position (Fig 4).

Although immunochemistry of lung of patients with idiopathic pulmonary fibrosis revealed abundant deposition of PN [15] we could not know for sure whether there was a fraction of deposited PN that was not solubilized by extraction with SDS and for that reason was not analyzed in our Western blotting. Therefore, we interrogated the spectra of peptides generated by digestion of SDS-insoluble fractions of experimental metastatic tumors [25] allowing carboxylation [+44] to be a modification. The fractions, which were generated from mice with xenografts of human colon cancer had been found to contain PN and Big-h3 derived from both human tumor and murine host [25]. A small number of carboxylated peptides were identified. These carboxyls mapped to both Asp and Glu. Most of the modified peptides were from tubulin and tubulin-associated proteins. A single β-carboxylated peptide from mouse βig-h3 was identified, carboxylated on Asp221. The only γ-carboxylated residue detected in a protein that would have passed through the endoplasmic reticulum was Glu230 of the murine fibrinogen β-chain. Although this analysis did not find the presence of γ-carboxylation on PN or βig-h3, data mining alone of spectra of peptides not specifically enriched for identification of γ-carboxylation cannot be taken as evidence that PN or βig-h3 was completely free of modifications because carboxylated sites could have been below the instrument’s limit of detection.

### Expression and analysis of recombinant PN from mammalian cells

**Fig 5:**
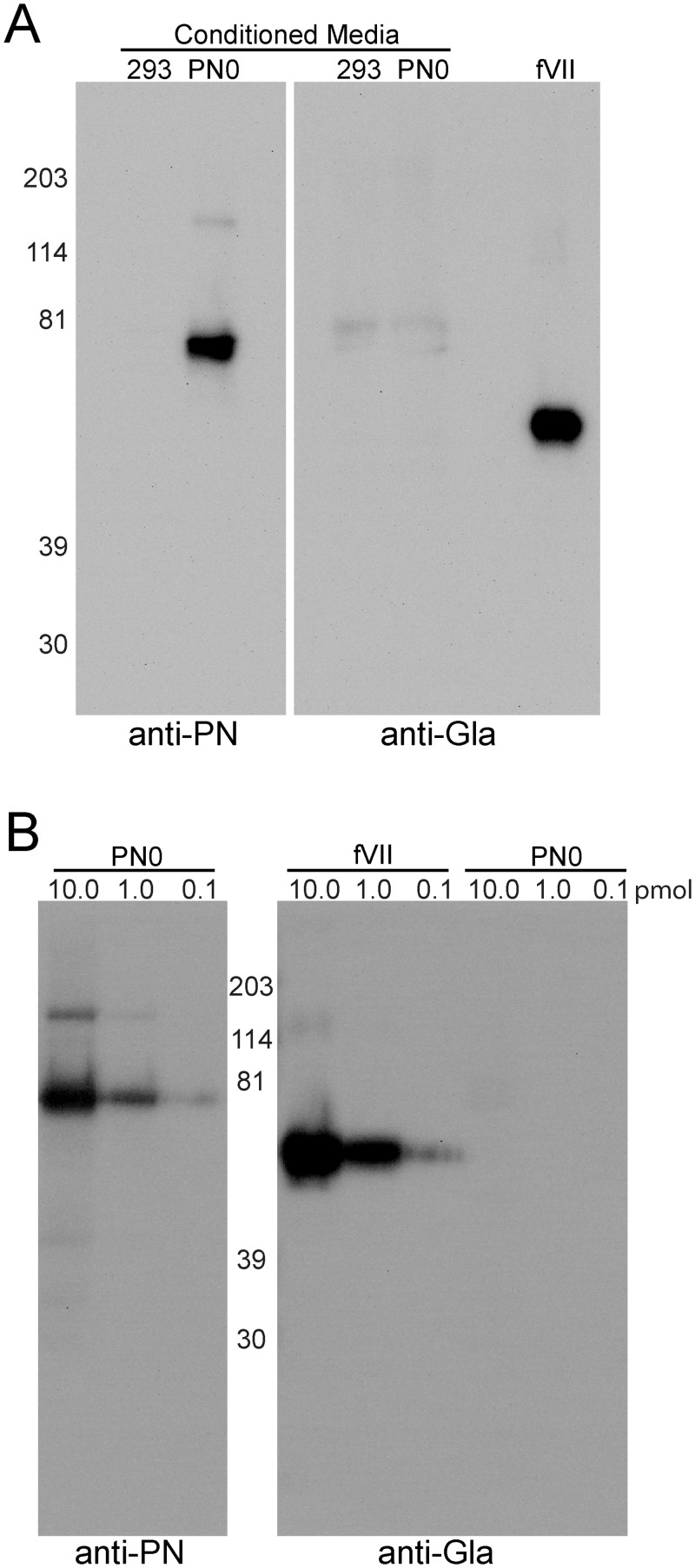
Recombinant Vitamin K-dependent coagulation factor VII produced in HEK293VKOR cells is γ-carboxylated whereas PN is not. (A) Conditioned media containing 10 μg/mL vitamin K from HEK293VKOR un-transfected (293) or transfected with PN0 (PN0), 30 μl, or 0.5 pmol of purified factor VII (fVII) were examined in Western blot. Mouse anti-PN (left) reacted strongly with media of cells transfected with PNO but not with control media. Both media were negative when probed with the mouse anti-Gla (right), but purified fVII reacted strongly. (B) Western blot analysis of purified fVII and PN0 expressed by HEK293VKOR cells in the presence of vitamin K. Varying amounts, 10.0, 1.0 and 0.1 pmol, of recombinant protein were probed with anti-PN (left) and anti-Gla (right). The anti-Gla antibody reacted with all inputs tested of fVII but not with PN.

**Fig 6:**
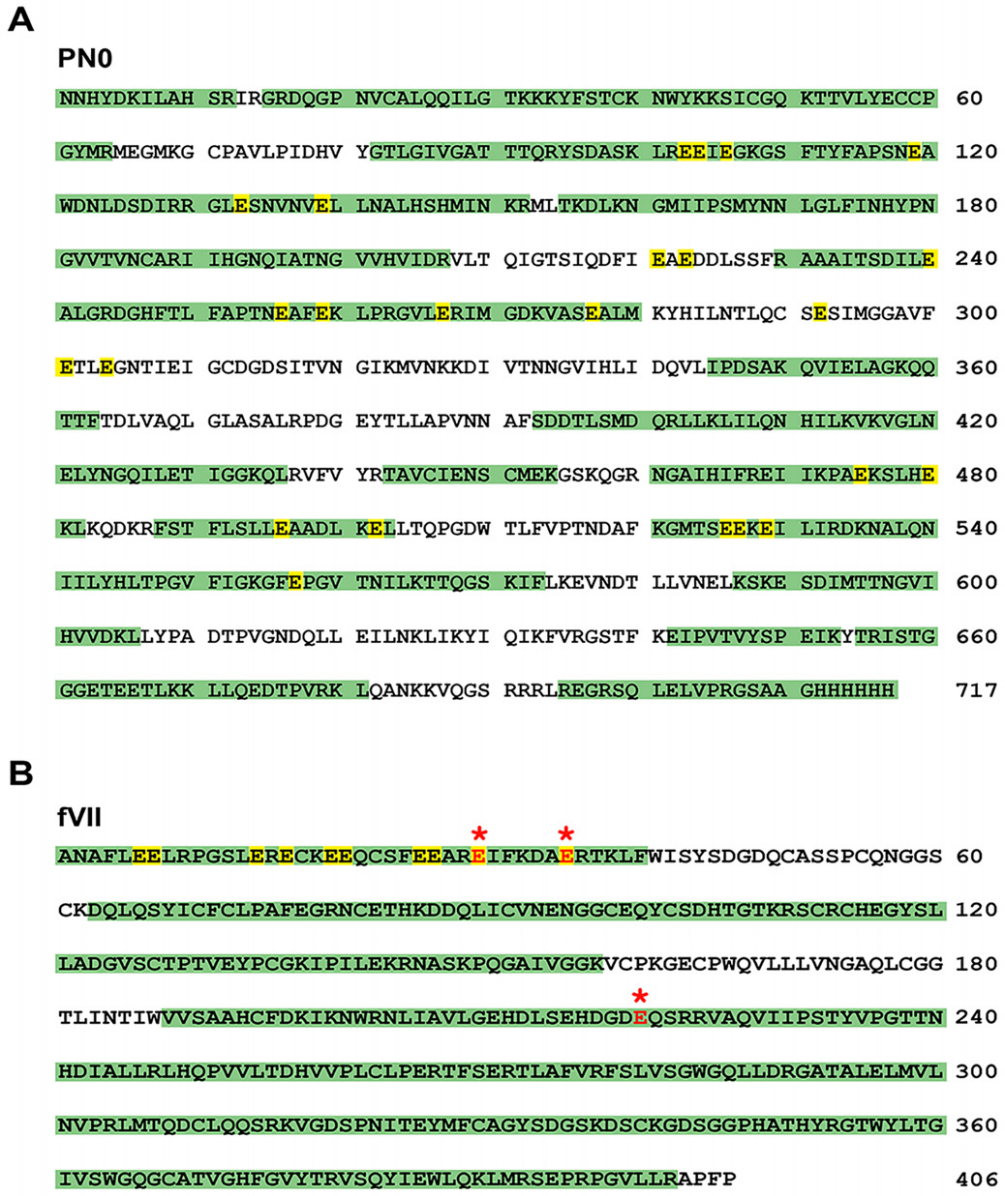
Mass Spec sequence coverage for PN0 and FVII. Combined ETD and AI-ETD sequence coverage of tryptic and chymotryptic digests of purified proteins. Mass spectrometry analysis obtained (A) 68% sequence coverage of PN0 and (B) 86% coverage of fVII. Identified peptides are shown in green, with Uniprot annotated γ-carboxylation sites highlighted in yellow. Experimentally verified γ-carboxylation sites for fVII are shown highlighted in red. Sequence and numbering are of the recombinantly expressed and secreted proteins and are therefore without the 21-residue signal sequence of PN or the 20-residue signal and 40-residue propeptide sequences of fVII.

Recombinant PN secreted into the media from HEK293 grown in medium containing vitamin K had previously been reported to react with anti-Gla mAb in Western blots [3]. In preliminary experiments we obtained positive results using anti-Gla at the 5 μg/ml concentration recommended by the manufacturer with PN secreted from insect SF9 or human HEK293 cells cultured both with or without vitamin K; contaminating proteins and the molecular weight markers reacted positively by Western blotting as well (S5A Fig). When we used a known vitamin K-dependent protein, blood coagulation factor VII (fVII), as a positive γ-carboxylation control, we found that the anti-Gla antibody could be diluted to concentrations at which reaction with contaminating proteins and proteins produced in insect cells in the absence of vitamin K (PN0 and FN70K) was minimized whereas reaction with fVII remained strong (S5B Fig). We then expressed PN and fVII in parallel in HEK293VKOR cells. These are HEK293 cells transfected with a plasmid encoding VKOR, which catalyzes both the reduction of vitamin K epoxide to vitamin K and the conversion of vitamin K to vitamin K hydroquinone, the rate-limiting step in producing vitamin K-dependent γ-carboxylation. VKOR overexpression has been shown to increase the fraction of recombinant protein that is γ-carboxylated [17, 30]. PN was detected by Western blotting in conditioned media of HEK293 cells co-transfected with PN0 and cultured in the presence of 10 μg/mL vitamin K, but nothing was secreted that reacted with anti-Gla antibody at a concentration of 0.5 μg/ml (Fig 5A). PN0 purified from serum-free conditioned media by Ni-chelate chromatography in the presence of 6M urea, which was added because it has been reported that denaturing of the protein is necessary for purification of the γ-carboxylated PN [3], also did not react with anti-Gla at 0.5 μg/ml (Fig 5B). The fVII positive control, in contrast, stained strongly with anti-Gla, 0.5 μg/ml (Fig 5A). The anti-Gla antibody, although it detects Gla residues irrespective of context, has been shown to vary in its ability to react with different γ-carboxylated proteins [18]. To address the protein-specific sensitivity issue, we compared fVII and PN over a 100-fold mass range (Fig 5B). Reactivity was found when as little as 0.1pmol fVII was analyzed whereas no reactivity was found with 10pmol PN. To detect Gla by a second method, we carried out mass spectrometry of tryptic and chymotryptic peptides of the two proteins with ETD and AI-ETD, techniques that have been optimized for identification of post translational modifications [27, 28]. Greater than 67% coverage of PN was achieved, including peptides with 19 of the 24 glutamates considered targets of γ-carboxylation (UniProt: Q15063-7). None were γ-carboxylated (Fig 6A). For fVII, γ-carboxylation was found for Glu89, Glu95 and Glu280 (Fig 6B and S6, S7, and S8 Figs).

**Fig 7:**
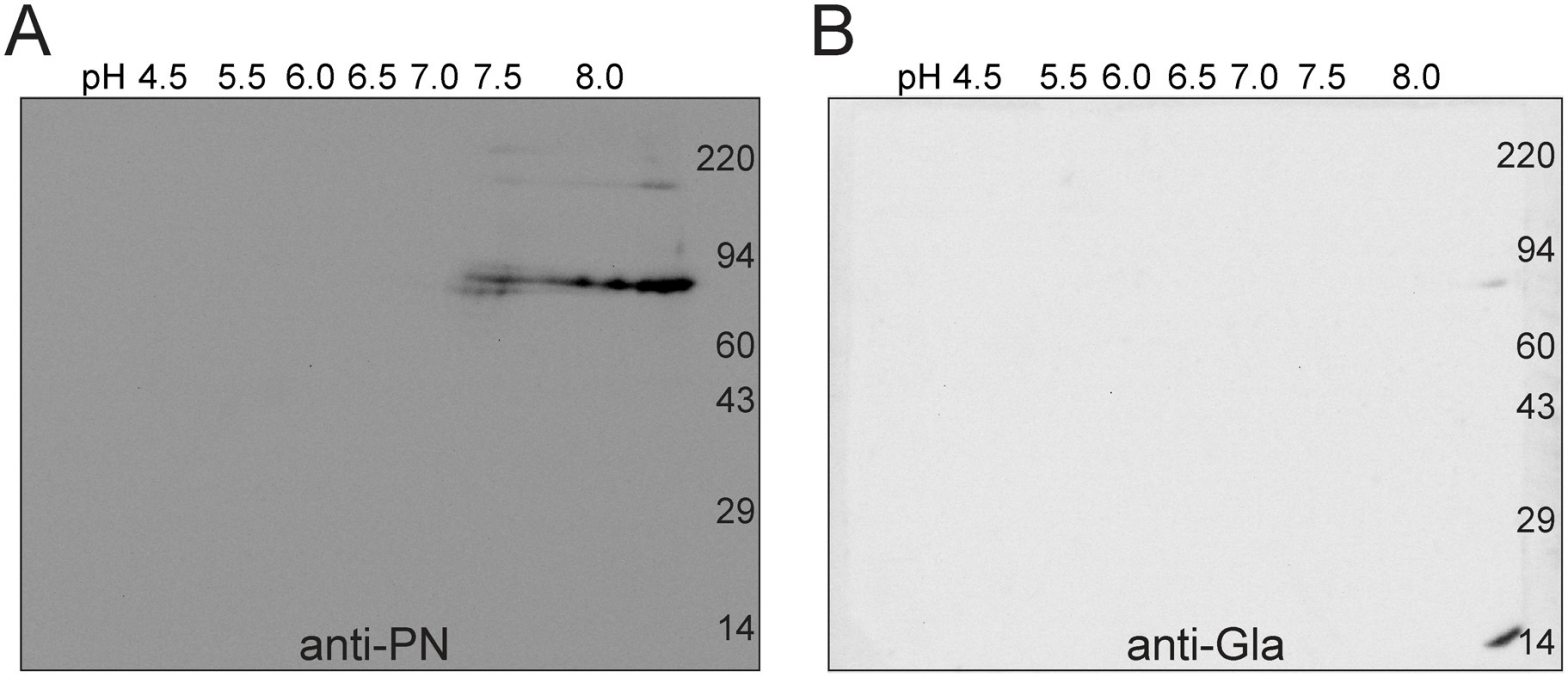
Recombinant PN expressed in HEK293VKOR cells has the expected pIs of non- γ-carboxylated protein. Purified PN0, 2.3 μg produced in the presence of 10 μg/mL vitamin K by HEK293VKOR cells was run on two-dimensional isoelectric focusing/electrophoresis and Western blotting gels. The parallel 8% gels were transferred to PVDF membranes and probed with (A) mouse anti-PN, or (B) mouse anti-Gla. Isoelectric points of PN positive spots ranged between pH 7.0 and > pH 8.0, consistent with unmodified protein. No anti-Gla-positive spots were detected.

Consistent with these findings, in two-dimensional electrophoresis, purified recombinant PN focused between pH 7.0 and >8 (Fig 7A), pIs similar to that found in the extracts from fibrotic lung, and appropriate for un-γ-carboxylated PN. Immunoblotting of the 2D gel of purified PN was negative with the anti-Gla antibody (Fig 7B).

## Discussion

UniProt annotates PN and βig-h3 to have up to 24 and 29 Gla modifications, respectively, which would easily make these two proteins the most extensively γ-carboxylated proteins in the proteome. γ-carboxylation profoundly changes the structure/function of the proteins it modifies, such that coagulation cascade factors II, X, IX, and VII; Proteins C, S, and Z; Gas6; osteocalcin; and matrix Gla protein [4, 5] are dependent on this modification for normal function. The initial aim of this study was to determine impact of γ-carboxylation state on PN structure-function. Studies of PN and βig-h3 are commonly done using recombinant proteins from bacteria, insect or mammalian cells cultured in the absence of vitamin K (i.e. expression systems lacking the ability to γ-carboxylate). If γ-carboxylated PN and βig-h3 are the functional form of these proteins *in vivo*, published and ongoing studies would be irrelevant. However, we were unable to obtain γ-carboxylated PN and, because the question of whether PN and βig-h3 are γ-carboxylated is important, now describe our attempts in detail. First, we found that PN extracted from fibrotic lung is not γ-carboxylated as assessed by its pI and lack of reactivity with antibody that recognizes Gla residues irrespective of context [18]. Second, we failed to make any detectible γ-carboxylated PN in a cell culture system that was optimized for production of γ-carboxylated fVII, which is considered to be one of the more difficult of the vitamin K-dependent blood coagulation factors to produce recombinantly in an active form [30]. Our studies were done with the same anti-Gla mAb that, along with acidic shift in two-dimensional gel electrophoresis, provided the most persuasive evidence for γ-carboxylation in the publication by Coutu et al. showing Gla modification of PN secreted from cultured mouse and human mesenchymal stromal cells and recombinant PN produced by HEK293 cells [3]. The immuno-blotting studies were complemented by ETD and AI-ETD mass spectrometry analyses of PN isolated from VKOR transfected 293 cells that failed to identify any γ-carboxylated peptides whereas analysis of a positive control (γ-carboxylated) sample of purified recombinant fVII localized three sites of γ-carboxylation. We did not investigate γ-carboxylation of βig-h3 to the same depth as we did PN, but in one-dimensional gel electrophoresis done in parallel with analysis of PN extracted from fibrotic lung, we found no evidence of modification. Also, our mass spectrometric analyses of PN are compatible with previous mass spectrometric analysis of βig-h3 extracted from cornea, which failed to reveal any post-translational modifications of either the whole protein or of peptides after digestion [31]. Finally, we were unable to find evidence for γ-carboxylated peptides from PN and βig-h3 in a database of mass spectra deposited from a study describing the composition of the SDS-insoluble fraction of tumors, which is known to contain PN and βig-h3[25]. It should be emphasized that the analyses of the mass spectrometric database and our mass spectrometry of PN and fVII, were of peptides that were not specifically enriched for γ-carboxylation. Further directed investigations would be needed to evaluate the lack of evidence for γ-carboxylation of fVII at the eight other previously described locations (Fig 6B) even though seven of them are reported to be reliably carboxylated in recombinant protein made by BHK cells [32] or the evidence of γ-carboxylation of fVII at Glu280, which has not been reported previously.

For most γ-carboxylated peptides or proteins, substrate recognition for the carboxylase resides in an N-terminal pro-sequence that is removed as the modified C-terminal peptide or protein matures in the endoplasmic reticulum and more distal secretory apparatus [33–35]. Matrix-Gla protein, which has a 16-residue γ-carboxylase recognition sequence embedded within a 77-residue protein [36], is an exception to the bipartite nature of γ-carboxylation and a model for the γ-carboxylase recognition of PN and βig-h3 as proposed by Coutu et al. [3]. There is no precedent, however, for γ-carboxylase having multiple recognition sequences embedded in a multi-modular protein as is proposed for PN and βig-h3 with putative recognition sequences identified in 3 of the 4 FAS1 modules [3]. Propeptides harboring *bona fide* γ-carboxylase recognition sequences have a propensity to form α-helices in 40% trifluoroethanol, and the prevailing view is that γ-carboxylation is determined by a chemical surface with a topology that is complementary to the surface of the propeptide binding site of the carboxylase [35]. However, the residues that allowed identification of putative γ-carboxylase recognition sequences in FAS1 modules [3] appear to be contributing importantly to tertiary structure rather than to surface topology. FAS1 modules have a distinctive global fold that was first revealed by the crystal structure of the third and fourth tandem FAS1 modules of *Drosophila* fasciclin-1[PDB ID code 1o70] [37] and shown to be true for the fourth FAS1 module of human βig-h3 by crystallography [PDB ID code 2VXP, not published], NMR [PDB ID code 2LTB] [38], and modeling [39]. An 11-residue sequence stretching from the β1-sheet to the α3-helix, which is the most conserved region of FAS1 modules in alignments of fasciclin-1 and βig-h3[39], overlaps with and includes 8 of the 16 residues in the putative γ-carboxylase recognition sequence. When one examines the structures of the fourth FAS1 module of βig-h3, side-chains of 3 of the 4 residues that are most conserved compared to known vertebrate γ-carboxylase recognition sequences [3], F540, A546, L550, and R555, are localized on the inner faces of the β-sheet and α-helix, with only R555 being solvent accessible [38]. A number of other side-chains also are oriented to the interior of the molecule. Side chains of the phenylalanines at the same position as F540 in the fasciclin-1 modules are, like F540, buried in a cavity surrounded by a 4-stranded β-sheet [39]. All-in-all, it is difficult to reconcile γ-carboxylation of FAS1 modules with what is known about γ-carboxylation of known substrates and the structure of FAS1 modules.

We can only speculate as to why our results contradict the findings of Coutu et al. One set of differences is with the characterization of recombinant PN made by 293 cells. Our system, which was geared toward expression of γ-carboxylated proteins and allowed production of γ-carboxylated fVII, produced PN that was not γ-carboxylated. The concentration of anti-Gla antibody used by Coutu et al. and us to test for γ-carboxylation status in Western blots is a potentially important variable because of the tendency of the antibody to react non-specifically when bands contained copious protein or the antibody was at a concentration > 1 ug/ml (S5 Fig). Another difference concerns the locations of PN and Gla-containing proteins on 2D gels. In the present study, we found that 3 γ-carboxylated proteins, fII, fIX, and protein S, migrated in the same region as PN on 1D SDS-PAGE and that fII was in our lung extracts by Western blotting. The major anti-Gla-staining band in the region co-migrated with fII and not with PN. In 2D gels, fII and fIX presumably account for some of the acidic Gla-positive, PN-negative spots of similar MW to that of PN. Coutu et al. [3] made no mention of whether the 2D gels were Western blotted with anti-PN or anti-βig-h3 antibodies, whether peptides from known γ-carboxylated proteins were found in the acidic region of the gel from which the PN- and βig-h3-derived peptides were identified, or whether any of the peptides from PN or βig-h3 contained Gla. Regarding why peptides from PN and βig-h3 could be generated from protein focusing to the acidic region [3], one possibility is that the different reagents used to prepare samples for the first dimension of the 2D separation, SDS by us and “Destreak rehydration buffer” by Coutu et al. [3], resulted in variable amounts of streaking. A second possibility is that PN or βig-h3 secreted from mesenchymal stromal or adenocarcinoma cells undergo loss of N- or C-terminal basic residues as a result of proteolysis, thus lowering the pI. Such has been demonstrated for βig-h3 from cornea [40]. A final possibility is that PN or βig-h3 carry other acidic modifications or are phosphorylated. PhosphoSitePlus (www.phosphosite.org) lists 10 residues in PN that have been identified as being phosphorylated in various phosphoproteomic screens.

## Supporting Information

S1 Fig SDS-PAGE of recombinant PN. PN0 separated into a monomer of ~76 kDa and lesser amounts of an apparent dimer of ~162 kDa without reduction and a monomer of ~78 kDa with reduction. PN was detected in Western blot using mouse anti-PN antibody.
(TIF)

S2 Fig Purity of fVII (fVII R152A) produced by HEK293VKOR cells. Silver stain analysis of 0.5 μg of recombinant fVII and plasma-derived (pd) fVII after separation by reducing SDS-PAGE. There is only one visible band in the recombinant fVII lane.
(TIF)

S3 Fig Detection of fII in fibrotic lung extracts. Fibrotic lung extracts (F) and purified fII (II) were probed with anti-sheep IgG after incubation with no primary antibodies (left) or sheep anti-fII (right). The fibrotic lung extract yielded a specific band that co-migrated with purified fII and non-specific bands of ~50 and ~20 kDa that presumably represents cross-reaction of anti-sheep IgG with human IgG in the lung extract.
(TIF)

S4 Fig Detection of PN in fibrotic lung extracts using polyclonal anti-PN antibodies. The same membrane used in Fig 3 was stripped and re-probed with polyclonal antibodies to PN. The pattern was similar to what was found with the mAb to PN except for the appearance of several low mw spots in the basic range, one low mw spot in the acidic range and that the”Y” shaped spot was not seen. All PN positive spots were negative with the anti-Gla antibody (Fig 3).
(TIF)

S5 Fig Non-specific binding and titration of anti-Gla antibody. (A) PN constructs PN0, PN19 (isoform 4) and PN1819 (isoform 3) were expressed in insect cells in the absence of vitamin K and immuno-blotted with 2.5 μg/mL anti-Gla. Both PN proteins and contaminants known to represent insect ferritin (24 and 20 kDa bands) stained positively. **(B)** Anti-Gla mAb at concentrations of 5, 2.5, 1, or 0.5 μg/mL was used in Western blot to probe PN0, FN70 (N-terminal portion of fibronectin) and fVII. PN0 and FN70 were expressed and purified from insect cells, an expression system which lacks the ability to γ-carboxylate. Factor VII was expressed in the presence of vitamin K by HEK293VKOR cells. The 10 pmol samples of PN0 and FN70 showed reaction when probed with 5 and 2.5 μg/mL anti-Gla indicating that high concentrations of antibody in combination with large amounts of protein result in non-specific staining that is lost when anti-Gla is diluted further. In contrast, all concentrations of anti-Gla resulted in heavy staining of the fVII positive control.
(TIF)

S6 Fig Annotated spectra for fVII Glu89. (A) Mass spectrum for localization of γ-carboxylation on Glu89. (B) Observed c and z ions are highlighted in red and blue, respectively.
(TIF)

S7 Fig Annotated spectra for fVII Glu95. (A) Mass spectrum for localization of γ-carboxylation on Glu95. (B) Observed c and z ions are highlighted in red and blue, respectively.
(TIF)

S8 Fig Annotated spectra for fVII Glu280. (A) Mass spectrum for localization of γ-carboxylation on Glu280. (B) Observed c and z ions are highlighted in red and blue, respectively.
(TIF)
