# Supplementary figures and images for: Absence of Vitamin K-Dependent γ-Carboxylation in Human Periostin Extracted from Fibrotic Lung or Secreted from a Cell Line Engineered to Optimize γ-Carboxylation
Source: PLoS One. 2015 Aug 14;10(8):e0135374. doi: 10.1371/journal.pone.0135374 (PMC4537219; doi:10.1371/journal.pone.0135374)

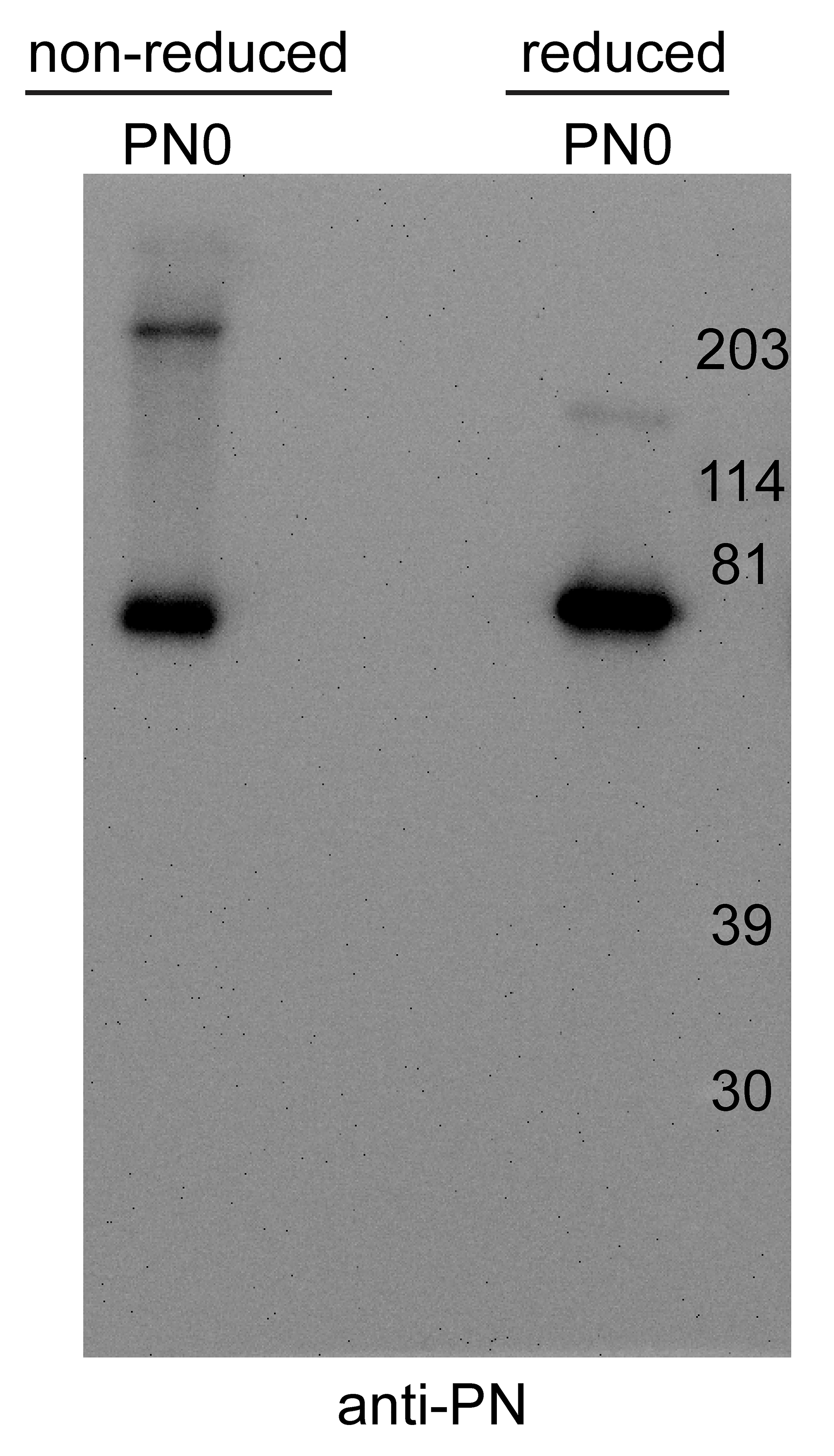

Supplement: S1 Fig — PN0 separated into a monomer of ~76 kDa and lesser amounts of an apparent dimer of ~162 kDa without reduction and a monomer of ~78 kDa with reduction. PN was detected in Western blot using mouse anti-PN antibody. (TIF) [file pone.0135374.s001.tif]

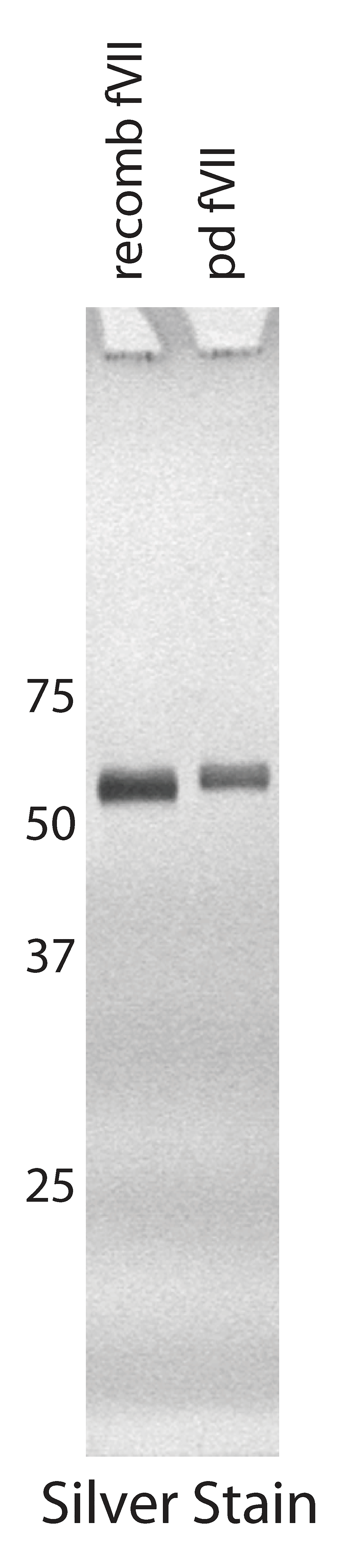

Supplement: S2 Fig — Silver stain analysis of 0.5 μg of recombinant fVII and plasma-derived (pd) fVII after separation by reducing SDS-PAGE. There is only one visible band in the recombinant fVII lane. (TIF) [file pone.0135374.s002.tif]

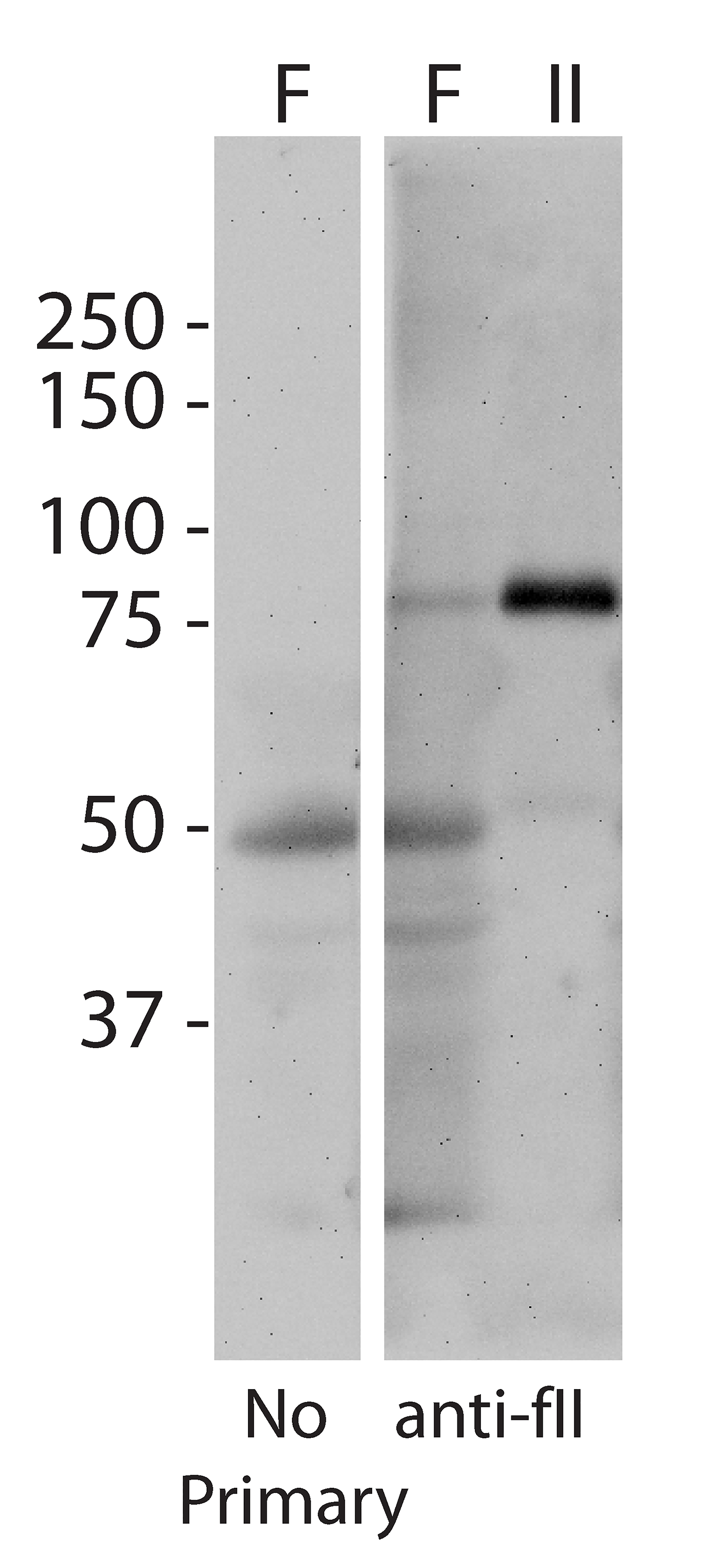

Supplement: S3 Fig — Fibrotic lung extracts (F) and purified fII (II) were probed with anti-sheep IgG after incubation with no primary antibodies (left) or sheep anti-fII (right). The fibrotic lung extract yielded a specific band that co-migrated with purified fII and non-specific bands of ~50 and ~20 kDa that presumably represents cross-reaction of anti-sheep IgG with human IgG in the lung extract. (TIF) [file pone.0135374.s003.tif]

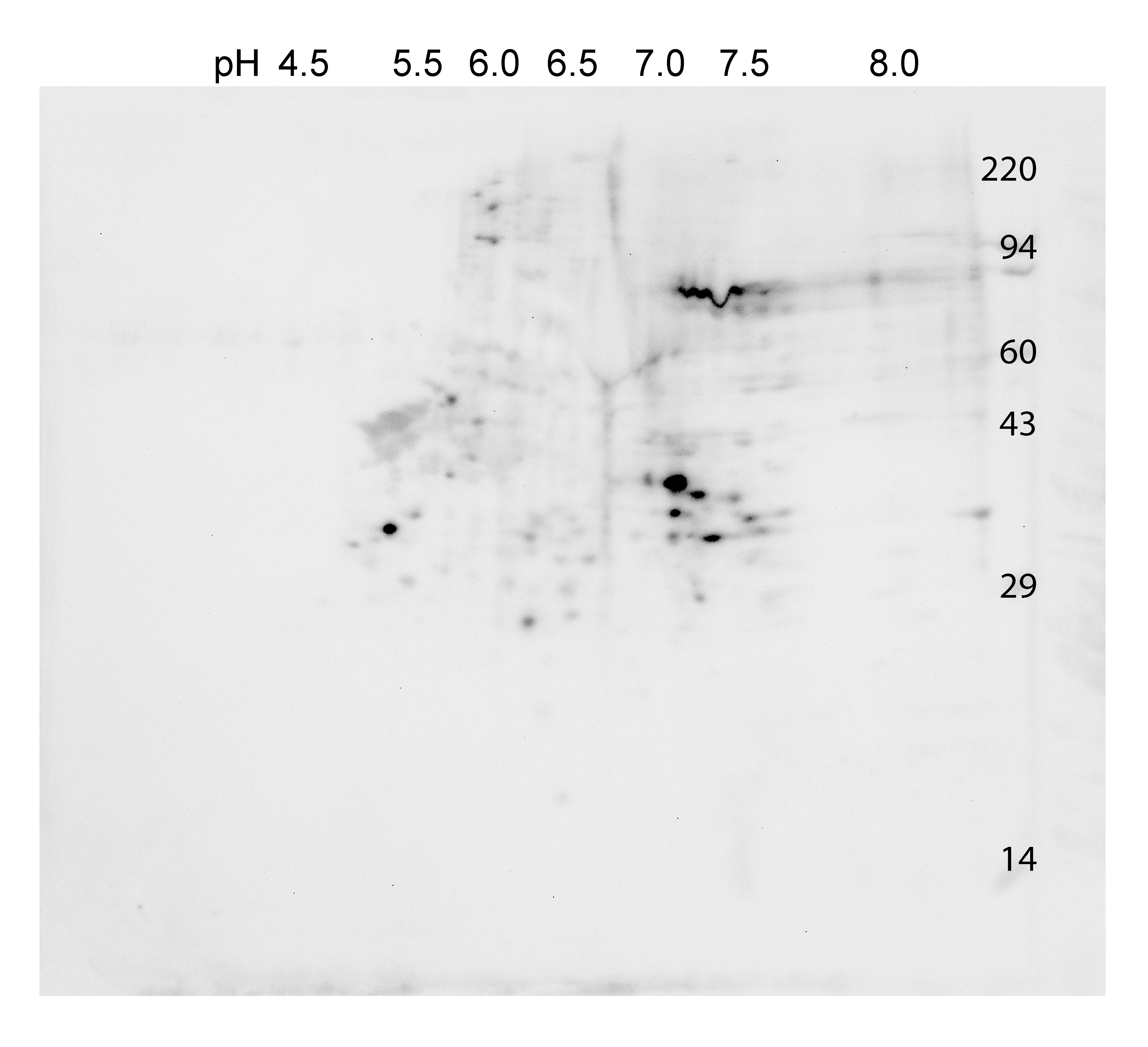

Supplement: S4 Fig — The same membrane used in Fig 3 was stripped and re-probed with polyclonal antibodies to PN. The pattern was similar to what was found with the mAb to PN except for the appearance of several low mw spots in the basic range, one low mw spot in the acidic range and that the”Y” shaped spot was not seen. All PN positive spots were negative with the anti-Gla antibody (Fig 3). (TIF) [file pone.0135374.s004.tif]

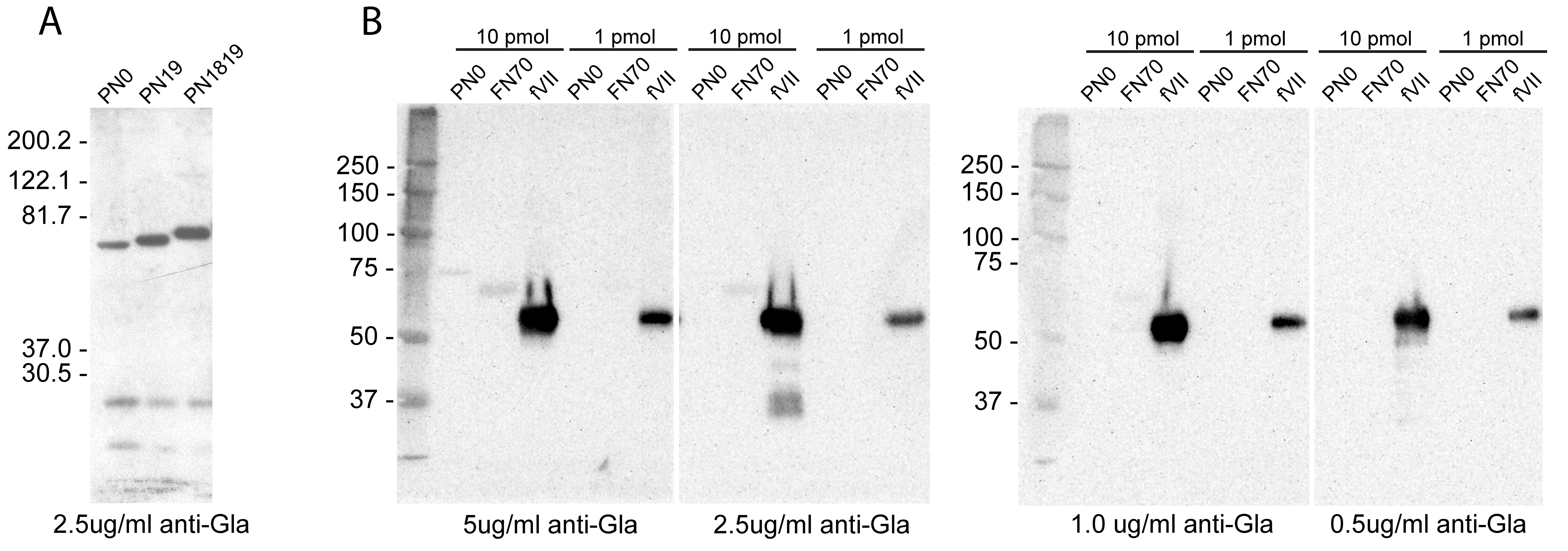

Supplement: S5 Fig — (A) PN constructs PN0, PN19 (isoform 4) and PN1819 (isoform 3) were expressed in insect cells in the absence of vitamin K and immuno-blotted with 2.5 μg/mL anti-Gla. Both PN proteins and contaminants known to represent insect ferritin (24 and 20 kDa bands) stained positively. (B) Anti-Gla mAb at concentrations of 5, 2.5, 1, or 0.5 μg/mL was used in Western blot to probe PN0, FN70 (N-terminal portion of fibronectin) and fVII. PN0 and FN70 were expressed and purified from insect cells, an expression system which lacks the ability to γ-carboxylate. Factor VII was expressed in the presence of vitamin K by HEK293VKOR cells. The 10 pmol samples of PN0 and FN70 showed reaction when probed with 5 and 2.5 μg/mL anti-Gla indicating that high concentrations of antibody in combination with large amounts of protein result in non-specific staining that is lost when anti-Gla is diluted further. In contrast, all concentrations of anti-Gla resulted in heavy staining of the fVII positive control. (TIF) [file pone.0135374.s005.tif]

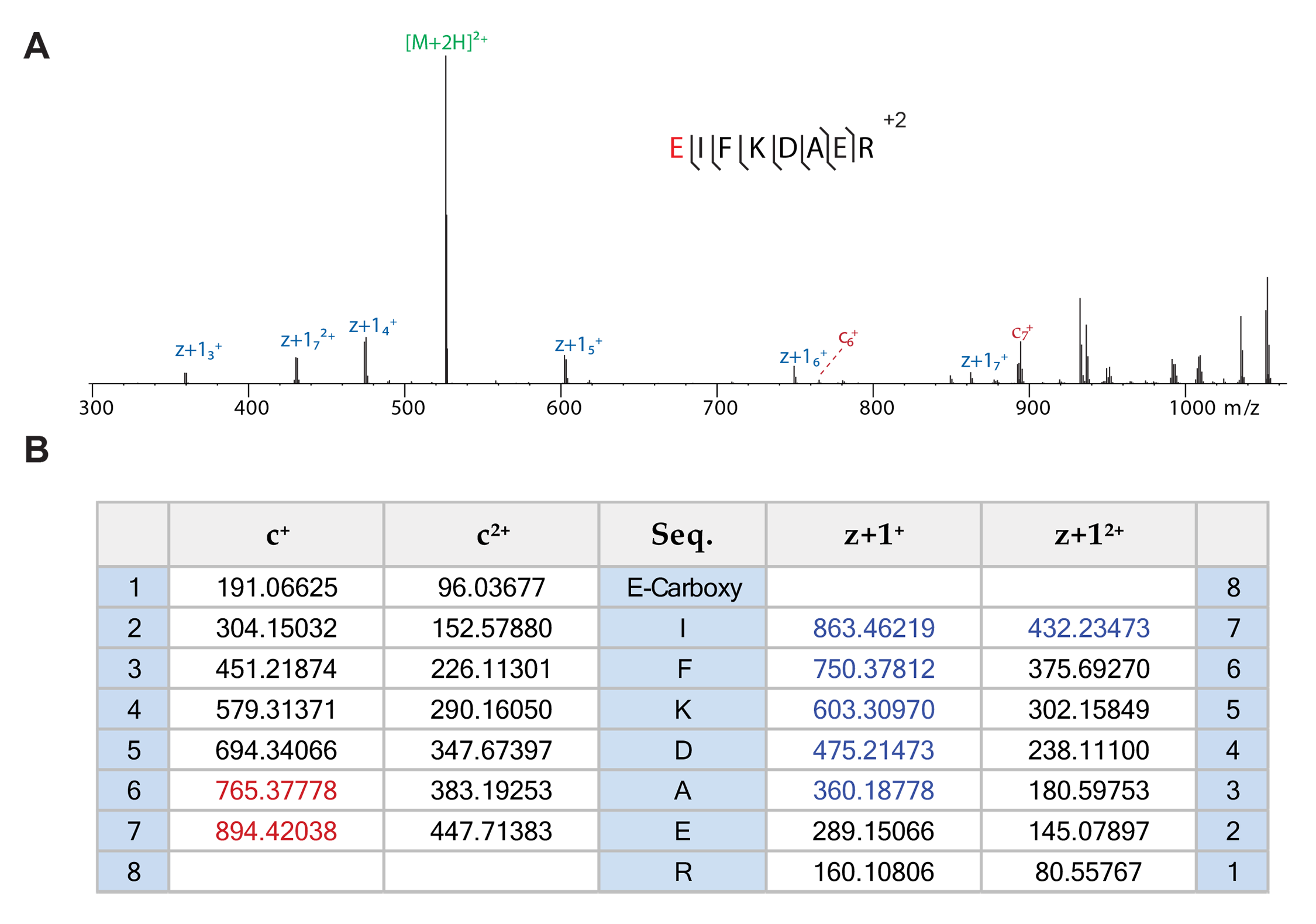

Supplement: S6 Fig — (A) Mass spectrum for localization of γ-carboxylation on Glu89. (B) Observed c and z ions are highlighted in red and blue, respectively. (TIF) [file pone.0135374.s006.tif]

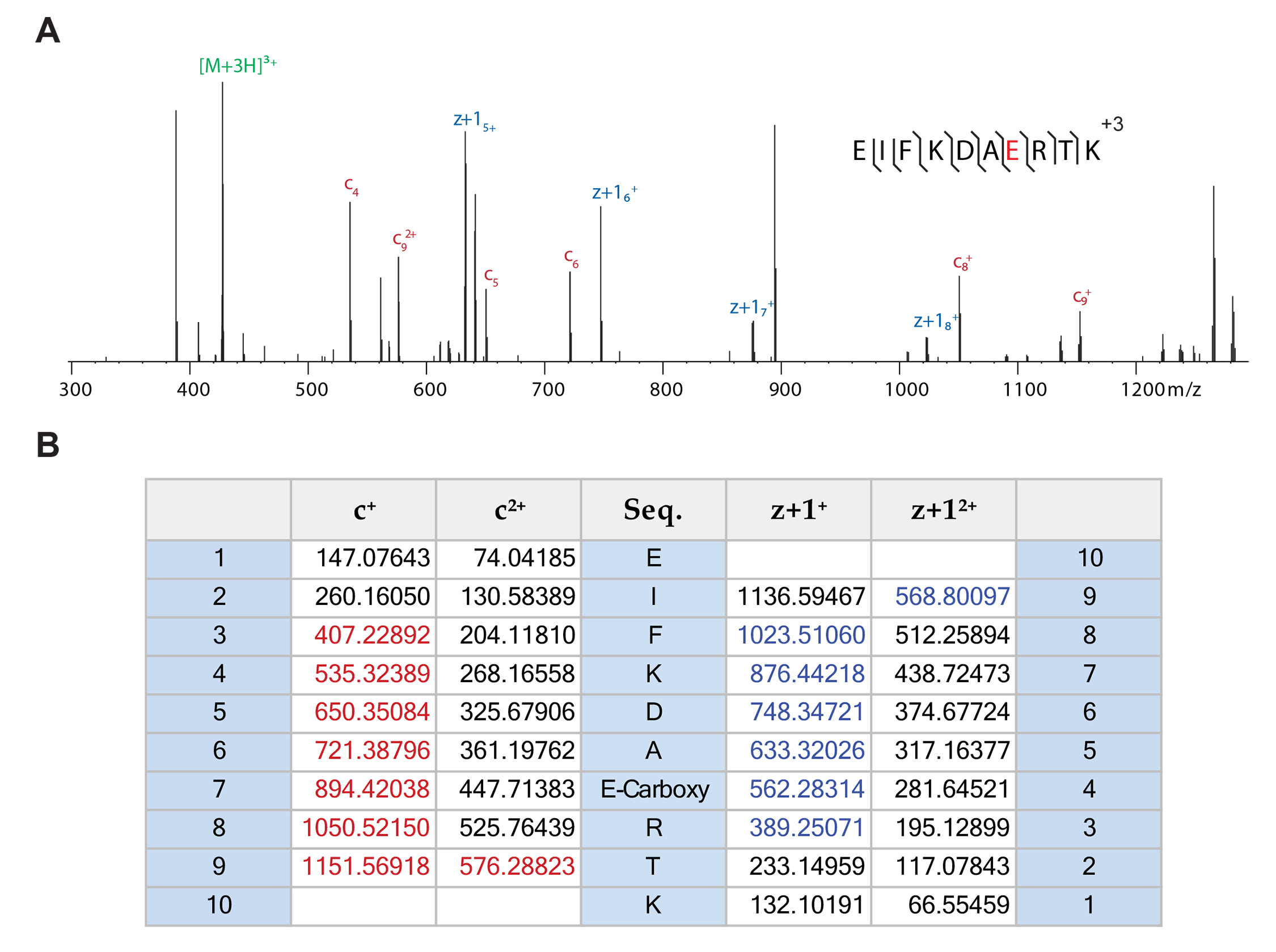

Supplement: S7 Fig — (A) Mass spectrum for localization of γ-carboxylation on Glu95. (B) Observed c and z ions are highlighted in red and blue, respectively. (TIF) [file pone.0135374.s007.tif]

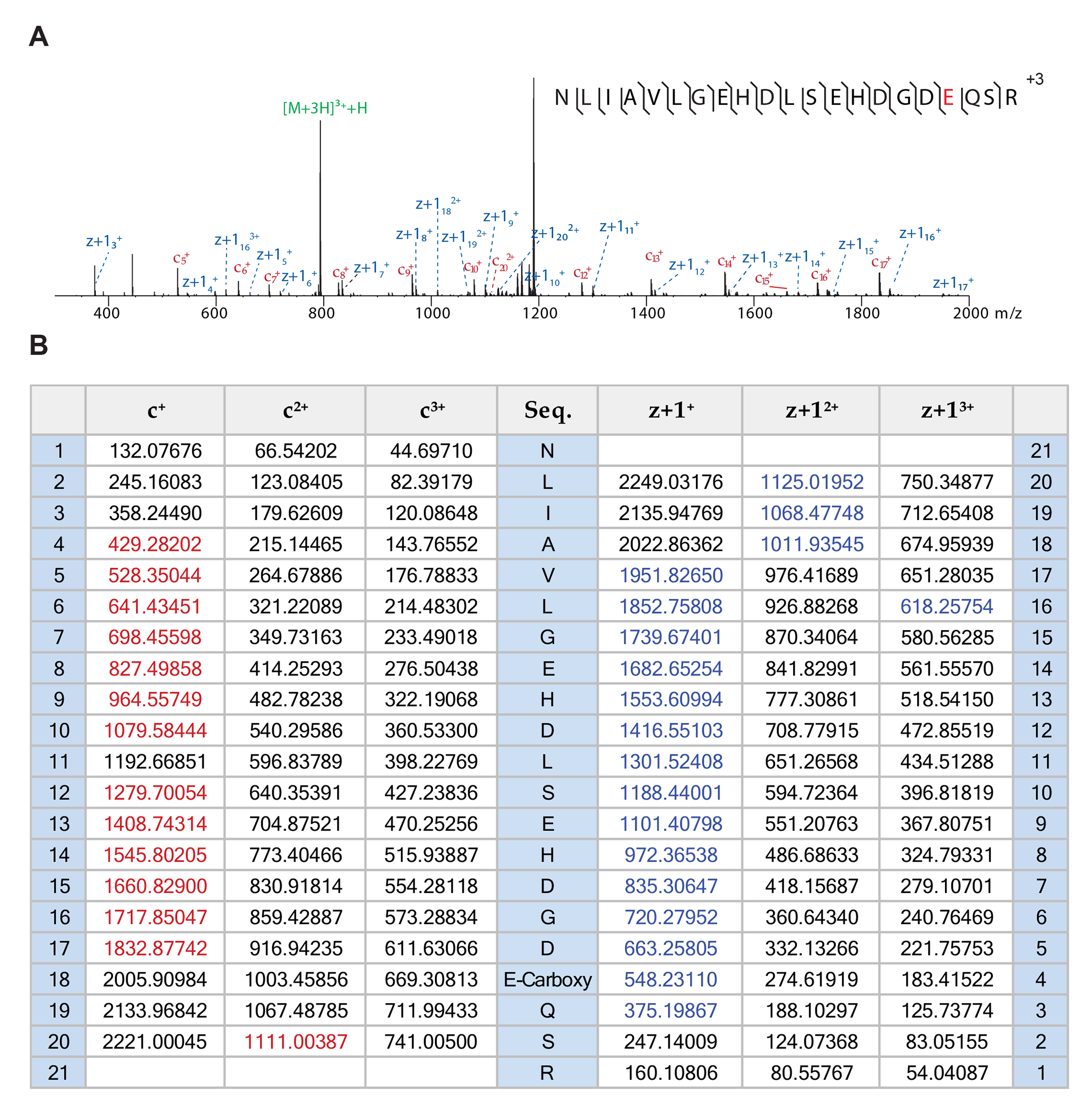

Supplement: S8 Fig — (A) Mass spectrum for localization of γ-carboxylation on Glu280. (B) Observed c and z ions are highlighted in red and blue, respectively. (TIF) [file pone.0135374.s008.tif]
